# Ultrasound of the Heel Improves Diagnosis—Tender Entheses in the Heel Region Rarely Corresponds to Inflammatory Enthesitis in Patients with Peripheral Spondyloarthritis

**DOI:** 10.3390/jcm11092325

**Published:** 2022-04-21

**Authors:** Sara Kamp Felbo, Mikkel Østergaard, Inge Juul Sørensen, Lene Terslev

**Affiliations:** 1 Copenhagen Center for Arthritis Research, Center for Rheumatology and Spine Diseases, Centre of Head and Orthopaedics, Rigshospitalet, 2600 Glostrup, Denmark; 2 Department of Clinical Medicine, University of Copenhagen, 2200 Copenhagen, Denmark

**Keywords:** ultrasound, enthesitis, spondyloarthritis

## Abstract

Enthesitis is a key pathology in spondyloarthritis (SpA), but diagnosis may be clinically challenging. The objective of this study was to investigate the prevalence of ultrasound enthesitis lesions in tender entheses in the heel region in patients with peripheral SpA. In 27 patients with tenderness upon palpation at the Achilles tendon or the plantar fascia insertion, ultrasound assessment of the affected enthesis was performed using greyscale and color Doppler mode. Images were evaluated using the Outcome Measures in Rheumatology (OMERACT) scoring system for enthesitis, scoring presence/absence of hypoechogenicity, thickening, calcifications/enthesophytes, and erosions, and color Doppler activity semi quantitatively from 0 to 3. A total enthesitis sum score was calculated. A second examiner scanned 10 patients for inter-reader reliability. Ultrasound signs of inflammatory enthesitis (thickening/hypoechogenicity and/or Doppler activity) were found in 48%, and 19% showed Doppler activity—all in the Achilles enthesis. Inflammatory pathologies other than enthesitis (e.g., tendinitis, arthritis, bursitis) were identified in 26% of tender heels. The ultrasound OMERACT scoring system for enthesitis lesions showed excellent intra- and inter-reader agreement in a clinical setting. In conclusion, less than 50% of clinically tender entheses are related to inflammatory enthesitis when assessed by ultrasound. Ultrasound is useful for diagnosing other pathologies that may explain tenderness in the area.

## 1. Introduction

Enthesitis, inflammation at the insertion of tendon, capsule, or ligament into the bone, is a key pathology in spondyloarthritis (SpA) diseases including psoriatic arthritis (PsA) [1,2,3]. It is defined as a key domain for assessing disease activity and response to treatment [4,5]. Objective findings of enthesis involvement may be scarce, as both clinical examination and biochemical parameters may be normal. Therefore, diagnosis and evaluation of disease activity typically rely on a characteristic medical history and patient-reported symptoms. Imaging has shown to be valuable in detecting enthesis involvement and is more sensitive than clinical examination for assessing inflammation at entheses [3,4,5]. Imaging may be used in both diagnosis and assessment of disease activity [6]. However, imaging studies of enthesitis have shown great discrepancies in the association between clinical findings and findings of inflammation by ultrasound where inflammatory changes may be seen in asymptomatic entheses and where symptomatic entheses may display no inflammatory changes [7,8].

The Outcome Measures in Rheumatology (OMERACT) Ultrasound Working Group has defined and validated the elementary lesions for enthesitis both in static images and in patients [1,2]. Enthesis thickening, hypoechogenicity, and Doppler activity are inflammatory lesions with Doppler activity indicating active inflammation. Bone erosions and enthesophytes/calcifications are structural lesions.

We have previously found a poor correlation between tender joints and signs of inflammation by ultrasound in patients with established psoriatic arthritis [9]. The aim of this study was to investigate the association between clinically tender Achilles and plantar fascia enthesis in patients with peripheral SpA and ultrasound signs of inflammation. Furthermore, we aimed to describe if other findings can explain clinically tender entheses.

## 2. Materials and Methods

### 2.1. Patients and Clinical Evaluation

Patients with SpA according to the ASAS criteria [10] who presented with tender Achilles tendon insertion or plantar fascia insertion at the calcaneus when evaluated by their treating rheumatologist were referred to the ultrasound clinic for assessment and potential inclusion in the study. If several tender entheses were present, the most tender on clinical examination was selected. The study was approved by the local ethics committee (J. no. H-16035123).

Clinical evaluation included counts of tender (68) and swollen (66) joints, tender entheses (according to Spondyloarthritis Research Consortium of Canada (SPARCC)), and a global evaluation of disease activity on a visual analog scale (VAS) by the patient’s usual rheumatologist and a measure of the level of C-reactive protein (CRP). Patients filled out the health assessment questionnaire (HAQ) and a global evaluation of disease activity (Pt global) and pain (Pt Pain) on a visual analog scale (VAS) (0–100).

### 2.2. Ultrasound Examination and Scoring

Ultrasound was performed the same day as the clinical examination, with a GE Logiq^®^ E9 machine, version R5 (Milwaukee, WI, USA) with a 6–15 MHz linear transducer, in greyscale (GS) and color Doppler (CD) modality. For CD, the frequency, pulse repetition frequency (PRF), and gain were set according to published guidelines [11] with a Doppler frequency of 7.5 MHz and a PRF of 0.4 MHz. The same settings were used for all patients. Tender entheses were examined in longitudinal and transverse planes and patients were positioned according to the European League Against Rheumatism (EULAR) guidelines [12] in the prone position and the foot in a neutral position. Enthesitis of the tender enthesis was scored using the OMERACT enthesitis scoring system for presence/absence of thickening, hypoechogenicity, calcifications/enthesophytes, and erosions and semi quantitatively 0–3 for CD activity ≤2 mm from the bony cortex [2,13]. Each lesion was scored separately and, subsequently, a sum score (0–7) was calculated by summing the binary scores (0/1) of thickening, hypoechogenicity, calcifications/enthesophytes and erosions, and the 0–3 score for color Doppler activity. All examinations were performed by one examiner (SKF, 5 years of musculoskeletal ultrasound experience) and 10 patients were also examined by a second examiner on the same day (LT, >20 years of experience) for inter-reader agreement. Stored images were re-read by the first examiner after 3 weeks for intra-reader agreement.

### 2.3. Statistics

Descriptive statistics are presented as numbers (percentages) for binary variables and as medians (interquartile ranges) for continuous variables. Inter- and intra-reader agreement, as well as the agreement between clinical and ultrasound findings of enthesitis, was evaluated using Cohen’s Kappa and prevalence and bias-adjusted Kappa (PABAK) [14] for binary outcomes, weighted Kappa (squared weights) for ordinal outcomes, and intraclass correlation coefficient (ICC) for sum scores. Kappa values of 0–0.20 were considered as slight agreement, 0.21–0.40 as fair, 0.41–0.60 as moderate, 0.61–0.80 as good, and 0.81–1.00 as excellent [15]. Findings at Achilles entheses vs. fascia plantaris were compared by Fischer’s exact test or Mann–Whitney U test, as appropriate (post hoc analyses). The significance level was set to *p* < 0.05. Statistical analyses were performed with R, version 3.6.1.

## 3. Results

### 3.1. Population Characteristics

Twenty-seven patients with peripheral SpA and tender entheses were included. Fourteen (52%) of the tender entheses were at the Achilles tendon insertion and 13 (48%) were at the plantar fascia insertion. PsA according to the CASPAR criteria could additionally be classified in 15 (56%) of the patients. Patients were a median (interquartile range) of 49 (38–56) years old, 59% were male and the median disease duration was 2 (0–6) years. The population characteristics for the cohort are shown in Table 1. The patients’ global pain scores (VAS 0–100) were high with a median (IQR) of 63 (41–73). We found no statistically significant differences in population characteristics between patients with PsA and other SpA (data not shown).

### 3.2. Ultrasound Findings and Agreement

**Figure 1:**
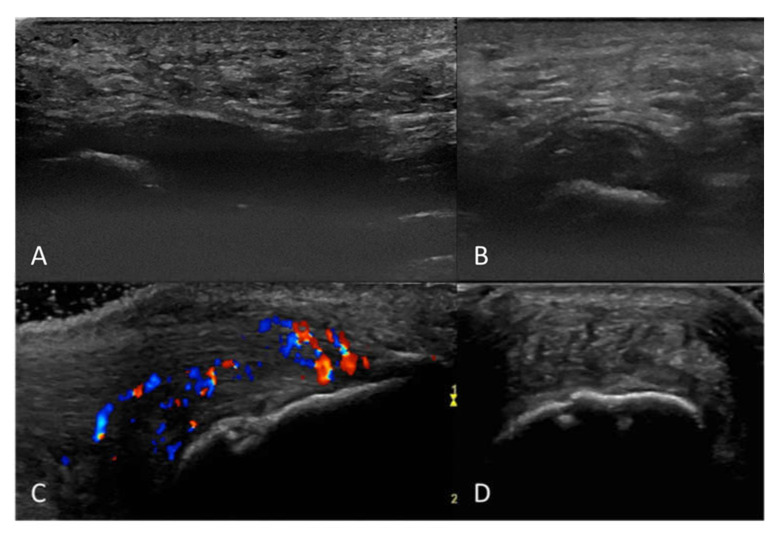
Greyscale images of the plantar fascia insertion with thickening and hypoechogenicity in longitudinal (**A**) and transverse (**B**) plane, and the Achilles tendon insertion with thickening, hypoechogenicity, color Doppler activity and enthesophyte in longitudinal plane (**C**) and greyscale image in transverse plane (**D**).

Ultrasound findings and the difference in findings between Achilles tendon and plantar fascia entheses are presented in Table 2 and image examples in Figure 1. One or more ultrasound signs of enthesitis (structural or inflammatory lesions) could be found in 19 (70%) of the tender entheses. Greyscale inflammatory ultrasound signs of enthesitis (thickening or hypoechogenicity) were found in 13 (48%) entheses, and 5 (19% of all entheses, 38% of entheses with greyscale signs of inflammation) showed CD activity. The most common inflammatory sign of enthesitis was thickening (13 (48%) of entheses), which was numerically somewhat more frequent at the plantar fascia (7 (54%)) compared to at the Achilles tendon (6 (43%)), while hypoechogenicity was seen in 12 (44%) of the entheses (6 (43%) Achilles entheses, 6 (46%) plantar fascia entheses). CD activity was seen only at the Achilles tendon (5 (36%) Achilles entheses, OR = 0, *p* = 0.04). Structural lesions were found in 44% of all entheses. Enthesophytes/calcifications were the most common lesion (12 (44%)) while erosions were more seldomly seen (4 (15%)). Both types of structural lesions (enthesophytes/calcifications and erosions) were statistically significantly more frequent at the Achilles tendon enthesis compared to the plantar fascia enthesis (OR for enthesophytes/calcifications 0.03, *p* < 0.001, for erosions 0.00, *p* = 0.098). No statistically significant differences were found in ultrasound lesions between patients with PsA and other types of SpA (data not shown).

**Figure 2:**
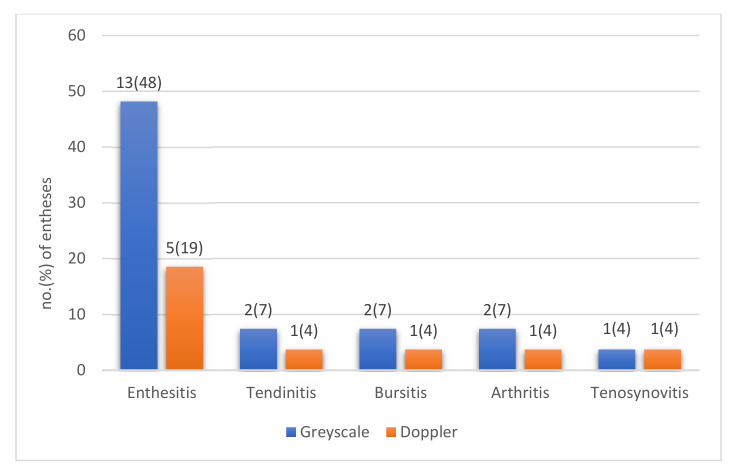
Ultrasound findings of inflammation in patients with spondyloarthritis (SpA) and tenderness at entheses in the heel region (*n* = 27) shown as no. (percentage) of tender entheses with presence of lesions in greyscale (blue) and color Doppler mode (orange).

In patients with tender entheses that did not show any ultrasound inflammatory signs of enthesitis (*n* = 14), other possible explanations for tenderness could be found in 7 (50%) (tendinitis (*n* = 2), arthritis in the subtalar joint (*n* = 2), tenosynovitis of the tibialis posterior tendon (*n* = 1), retrocalcaneal bursitis (*n* = 2)). Findings in greyscale and CD are shown in Figure 2.

Agreements between tenderness versus any ultrasound signs of enthesitis versus ultrasound inflammatory signs of enthesitis versus Doppler activity in the enthesis and ultimately versus ultrasound enthesitis OR other explanatory pathology are shown in Table 3. Overall, an inflammatory explanation for tenderness (enthesitis or other inflammatory pathology) could be found by ultrasound in 20 (74%) of the heels with tender entheses.

Intra- and inter-reader agreements are shown in Table 4. Intra-reader agreement for all individual lesions was excellent (Cohens Kappa 0.93–1.00, PABAK 0.93–1.00), as was ICC for sum scores (0.99 (0.98–1.00)). Inter-reader agreement was marginally lower both for individual lesions (Cohen’s Kappa 0–1, PABAK 0.8–1.0) and for sum scores (ICC 0.98 (0.93–0.99)).

## 4. Discussion

In this cross-sectional study of tender heel entheses in 27 patients with SpA, only 48% were found to be related to ultrasound signs of inflammatory enthesitis and only 19% had signs of Doppler activity (active inflammation—all in the Achilles enthesis). The lack of Doppler findings at the plantar fascia insertion may be explained by attenuation of the ultrasound by the heel fat pad, limiting the ability to detect Doppler activity. Therefore, greyscale signs of inflammation are of more importance here.

The most prevalent enthesitis lesion was thickening/hypoechogenicity at the Achilles insertion and thickening at the plantar fascia insertion. In some of the tender entheses without ultrasound signs of enthesitis, we could identify other origins of the pain than enthesitis (subtalar synovitis, tenosynovitis and retrocalcaneal bursitis). Thus, this study underlines the value of ultrasound for establishing the origin of pain around the heel in patients with SpA, as the treatment options for enthesitis in SpA are different than for arthritis. Therefore, the presence or absence of different pathologies may impact clinical decision-making, optimizing the outcome for the individual patient.

The poor association between clinical symptoms at the enthesis level and objective signs of inflammatory activity by ultrasound has also been demonstrated by Michelsen et al. [16] who found a lack of association between clinical and US signs of enthesitis in a cohort of PsA patients focusing only on the Achilles tendon. They also found that the signs of active inflammation and structural changes were similar in patients with and without tender enthesis, highlighting that clinical examination may have limited value as compared to ultrasound evaluation.

Structural lesions were found in 44% of the symptomatic entheses, with a much higher frequency in Achilles compared to plantar fascia entheses, and with calcifications/enthesophytes being the predominant findings. This is in line with the findings by Seven et al. [7] who found structural lesions to be the predominant lesion of lower limb entheses in an axial cohort of SpA patients initiating TNF alpha blocker treatment, irrespective of tenderness. They also found that these structural changes had no sensitivity to change. Enthesophytes are also a common finding in healthy controls and appear to be increasingly frequent with age [17]. The structural ultrasound lesions, therefore, seem to be unrelated to clinical entheses tenderness, and this challenges the usefulness of ultrasound structural lesions in the assessment of tender entheses. A longitudinal investigation of the development of these lesions in patients with newly diagnosed SpA and control groups could, however, be interesting.

Strengths of our study are the use of the OMERACT consensus-based and validated ultrasound enthesitis definitions and scoring system and the excellent inter- and intra-reader agreements of the scoring system. A limitation is the small sample size, for both the study cohort and the inter-reader analyses, with only 10 patients included for the latter analyses. Another limitation is the lack of blinding and control group.

In conclusion, less than half of clinically tender entheses in the heel region had ultrasound signs of inflammatory enthesitis (greyscale) and 19% were Doppler positive. Ultrasound was able to identify other pathologies as the origin of the heel pain. Ultrasound assessment of tender entheses is helpful for correct diagnosis and treatment decisions.

## Figures and Tables

**Table 1:** Population characteristics for all patients and patients with tender Achilles and plantar fascia entheses, respectively.

	All *n* = 27	Achilles *n* = 14	Fascia Plantaris *n* = 13	Difference	
	No/Median (%/IQR)	No/Median (%/IQR)	No/Median (%/IQR)	OR (95% CI)/ Difference in Medians (95% CI) ^1^	*p*
Age (years)	49 (38–56)	50 (39–57)	44 (37–52)	3 (−9–14)	0.56
Sex (male)	16 (59)	8 (57)	8 (62)	1.2 (0.2–7.3)	1
PsA	15 (56)	8 (57)	7 (54)	1.1 (0.2–6.7)	1
Enthesis—Achilles	14 (52)	14 (100)	0 (0)	-	-
Enthesis—Plantar fascia	13 (48)	0 (0)	13 (100)	-	-
Disease duration (years)	2 (0.25–6)	1 (0–6)	2 (1–6)	−1 (−4–3)	0.42
CRP (mg/L)	3.5 (1.5–6.8)	4.4 (1.6–7.8)	2.8 (1.8–4.7)	1.0 (−1.8–4.5)	0.54
TJC (0–68)	1 (0–11)	2 (0–11)	1 (0–8)	0 (−2–4)	0.75
SJC (0–66)	0 (0–0)	0 (0–0)	0 (0–0)	0 (0–0)	0.64
SPARCC (0–16)	2 (1–4)	3 (1–4)	2 (1–2)	0 (−1–2)	0.40
DAS28-CRP	2.5 (2.1–3.0)	2.3 (2–2.8)	2.6 (2.3–3.3)	−0.3 (−0.8–0.3)	0.18
Physician global VAS (0–100)	27 (14–42)	30 (23–70)	17 (12–36)	13 (−5–36)	0.15
HAQ (0–3)	0.88 (0.50–1.20)	0.75 (0.38–1.38)	0.88 (0.75–1.13)	−0.25 (−0.75–0.38)	0.38
Pt. global VAS (0–100)	72 (52–78)	71 (19–78)	72 (53–78)	−4 (−33–13)	0.68
Pt. pain VAS (0–100)	63 (41–73)	65 (38–79)	59 (47–67)	5 (−25–21)	0.70

CI: Confidence Interval, CRP: C-reactive protein, DAS28-CRP: Disease Activity Score in 28 joints using C-reactive protein, HAQ: Health Assessment Questionnaire, IQR: Interquartile range, OR: Odd ratio, Pt.: Patient, PsA: Psoriatic arthritis, SJC: Swollen Joint Count, SPARCC: Spondyloarthritis Research Consortium of Canada enthesitis index, TJC: Tender Joint Count, VAS: Visual Analogue Scale. ^1^ OR (95% CI) by Fisher’s exact test for binary variables, difference in medians (95% CI) by Mann–Whitney U test for continuous variables.

**Table 2:** Ultrasound findings and difference between findings at the Achilles and plantar fascia entheses.

	All *n* = 27	Achilles *n* = 14	Fascia Plantaris *n* = 13	Difference	
	No/Median (%/IQR)	No/Median (%/IQR)	No/Median (%/IQR)	OR (95% CI)/ Difference in Medians (95% CI) ^1^	*p*
**Elementary lesions**					
Thickening	13 (48)	6 (43)	7 (54)	1.5 (0.3–9.2)	0.71
Hypoechogenicity	12 (44)	6 (43)	6 (46)	1.1 (0.2–6.7)	1
Calcifications/Enthesophytes	12 (44)	11 (79)	1 (8)	0.0 (0.0–0.3)	<0.001
Erosions	4 (15)	4 (29)	0 (0)	0.0 (0.0–1.5)	0.10
CD (presence)	5 (19)	5 (36)	0 (0)	0.0 (0.0–1.0)	0.04
CD grade (positive only)	2 (2–2)	2 (2-2)	NA	-	-
**Combined lesions**					
Any inflammatory lesion ^2^	13 (48)	6 (43)	7 (54)	1.5 (0.3–9.2)	0.71
Any structural lesion ^2^	12 (44)	11 (79)	1 (8)	0.0 (0.0–0.3)	<0.001
Any inflammatory AND any structural lesion ^2^	6 (22)	5 (36)	1 (8)	0.2 (0.0–1.8)	0.17
Any inflammatory OR any structural lesion ^2^	19 (70)	12 (86)	7 (54)	0.2 (0.0–1.6)	0.10
**Sum-score**					
Sum-score (0–7) ^3^	1 (0.0–2.5)	1.5 (1–4)	1 (0–2)	1.0 (0.0–3.0)	0.09

CD: Color Doppler, CI: Confidence interval, IQR: Interquartile range, OR: Odds Ratio. ^1^ OR (95% CI) by Fisher’s exact test for binary variables, difference in medians (95% CI) by Mann–Whitney U test for continuous variables. ^2^ Inflammatory lesion = thickened and/or hypoechogenic with/without CD activity. Structural lesion = enthesophytes and/or erosions. ^3^ Sum of binary scores (0/1) of thickening, hypoechogenicity, calcifications/enthesophytes and erosions, and 0–3 score for color Doppler activity.

**Table 3:** Agreement between entheseal tenderness and ultrasound signs of enthesitis.

	PEA	κ	PABAK
Tenderness vs. any US sign of enthesitis (inflammatory ^1^ or structural ^2^)	70	0	0.41
Tenderness vs. any US inflammatory sign of enthesitis ^1^	48	0	−0.04
Tenderness vs. US inflammatory enthesitis ^1^ with Doppler activity	19	0	−0.63
Tenderness vs. any US inflammatory signs of enthesitis ^1^ OR other explanatory pathology	70	0	0.41

κ: Cohens Kappa, PABAK: Prevalence Adjusted Bias Adjusted Kappa, PEA: Percent Exact Agreement, US: ultrasound. ^1^ Hypoechogenicity and/or thickening. ^2^ Enthesophytes/calcifications and/or erosions.

**Table 4:** Intra- and inter-reader agreements of ultrasound elementary lesions of enthesitis and sum score.

	Intrareader (*n* = 27)				Interreader (*n* = 10)			
	Prev. (%)	PEA (%)	κ	PABAK	Prev. (%)	PEA (%)	κ	PABAK
Thickened	48	100	1	1	20	100	1	1
Hypoechogenicity	46	96	0.93	0.93	5	90	0	0.8
Erosions	15	100	1	1	20	100	1	1
Enthesophytes/Calcifications	44	100	1	1	60	100	1	1
Color Doppler presence	19	100	1	1	10	100	1	1
Color Doppler grade (0–3)	NA	96	0.97	NA	NA	100	1	NA
Inflammation ^1^ yes/no	48	100	1	1	20	100	1	1
Structural ^2^ yes/no	44	100	1	1	60	100	1	1
	**ICC (95% CI)**				**ICC (95% CI)**			
Ultrasound lesion Sum-score ^3^	0.99 (0.98–1.00)				0.98 (0.93–0.99)			

CI: Confidence Interval, ICC: Intraclass Correlation, κ: Cohens Kappa, PABAK: Prevalence Adjusted Bias Adjusted Kappa, PEA: Percent Exact Agreement, Prev.: Mean prevalence of the two reads. ^1^ Inflammation = hypoechogenicity and/or thickening with/without Color Doppler activity. ^2^ Structural = enthesophytes and/or erosions. ^3^ Sum of the binary scores (0/1) of thickening, hypoechogenicity, calcifications/enthesophytes and erosions, and the 0–3 score for color Doppler activity.

## Data Availability

The datasets used and/or analyzed during the current study are available from the corresponding author on reasonable request.
